# Analyzing Twitter Conversation on Genome-Edited Foods and Their Labeling in Japan

**DOI:** 10.3389/fpls.2020.535764

**Published:** 2020-10-22

**Authors:** Yutaka Tabei, Sachiko Shimura, Yeondae Kwon, Shizu Itaka, Nobuko Fukino

**Affiliations:** ^1^Strategic Planning Headquarters, National Agriculture and Food Research Organization, Tsukuba, Japan; ^2^Research Center for Agricultural Information Technology, National Agriculture and Food Research Organization, Tokyo, Japan; ^3^Institute of Agrobiological Sciences, National Agriculture and Food Research Organization, Tsukuba, Japan; ^4^Faculty of Science and Technology, Tokyo University of Science, Noda, Japan

**Keywords:** genome editing, SNS, co-occurrence network, sentiment analysis, public acceptance

## Abstract

In recent years, the research and development of genome editing technology have been progressing rapidly, and the commercial use of genome-edited soybean started in the United States in 2019. A preceding study’s results found that there is public concern with regard to the safety of high-tech foods, such as genetically modified foods and genome-edited foods. Twitter, one of the most popular social networks, allows users to post their opinions instantaneously, making it an extremely useful tool to collect what people are actually saying online in a timely manner. Therefore, it was used for collecting data on the users’ concerns with and expectations of high-tech foods. This study collected and analyzed Twitter data on genome-edited foods and their labeling from May 25 to October 15 in 2019. Of 14,066 unique user IDs, 94.9% posted 5 or less tweets, whereas 64.8% tweeted only once, indicating that the majority of users who tweeted on this issue are not as intense, as they posted tweets consistently. After a process of refining, there were 28,722 tweets, of which 2,536 tweets (8.8%) were original, 326 (1.1%) were replies, and 25,860 (90%) were retweets. The numbers of tweets increased in response to government announcements and news content in the media. A total of six prominent peaks were detected during the investigation period, proving that Twitter could serve as a tool for monitoring degree of users’ interests in real time. The co-occurrence network of original and reply tweets provided different words from various tweets that appeared with a certain frequency. However, the network derived from all tweets seemed to concentrate on words from specific tweets with negative overtones. As a result of sentiment analysis, 54.5% to 62.8% tweets were negative about genome-edited food and the labeling policy of the Consumer Affairs Agency, respectively, indicating a strong demand for mandatory labeling. These findings are expected to contribute to the communication strategy of genome-edited foods toward social implementation by government officers and science communicators.

## Introduction

Agricultural production is facing new challenges due to the increasing world population, global climate change, and change in consumers’ attitudes. To respond to these changes, new breeding technology, such as genome editing, is highly anticipated (Lusser et al., 2011; Yin et al., 2017; Ghogare et al., 2019). Genome editing is an innovative technology that may accelerate breeding by pinpointing and changing specific gene(s) and nucleotide(s) related to yield, biotic and abiotic stress tolerance, nutritional components, growth, and other factors (Dale et al., 2017; Abe et al., 2019; Gomez et al., 2019; Romero and Gatica-Arias, 2019). Genome editing has been used to modify many crops, fish, livestock, and other living organisms; the first commercialized genome-edited crop was the Calyno^TM^ high-oleic soybean^1^, which has been cultivated in the United States since 2019. In Japan, the Cross-ministerial Strategic Innovation Promotion Program (SIP) of the Cabinet Office, which started in 2014, and other projects financed by the government have been the main supporters of the development of agricultural and animal products through genome editing technology. Examples include tomatoes rich in gamma-aminobutyric acid (GABA) (Nonaka et al., 2017), potatoes with significantly reduced natural toxins (solanine and chaconine) (Sawai et al., 2014), wheat with altered dormancy (Abe et al., 2019), red sea bream (Kishimoto et al., 2018) and Japanese pufferfish (Kuroyanagi et al., 2018), which have been modified to grow rapidly with more edible meat.

The social implementation of genome-edited products requires three main conditions: (i) the government’s handling policy, (ii) intellectual property rights, and (iii) public acceptance (Tabei, 2019a). Japan has just established basic handling systems of genome-edited organisms and foods. This means that if the editing process involves only changes in the genetic code within the range of natural mutation, and no foreign DNA sequence exists in the edited organism’s genome, the derived genome-edited food (hereinafter “genome-edited food”) may be exempt from regulation as genetically modified (GM) foods under the Food Sanitation Law (Tabei, 2019b). Notification, not safety assessment, is required for genome-edited foods before commercialization. Intellectual property rights cannot be discussed here because it largely depends on individual conditions. Public acceptance is considered essential for its implementation (Araki and Ishii, 2015), and a major premise here is product development that benefits both producers and consumers. A preceding study’s results found that there is some public concern with regard to the safety of high-tech foods produced using new breeding techniques (Malyska et al., 2016). For genome-edited crops, close communication of risks and benefits has been proposed for future social integration (Ishii and Araki, 2016). To achieve this, understanding the opinions, concerns, and expectations of consumers is considered important.

Generally, questionnaire surveys have been carried out to identify consumer interests and concerns. This method has the great advantage of garnering more information through answers to detailed questions, along with the information about the respondent’s background. On the contrary, this method comes with some shortfalls. First, it is difficult to collect the ideal quantity of opinions in a timely manner and therefore analyze it in response to changing social situations. Second, it has been suggested that surveys do not capture the conversational or hierarchical nature of public opinion formation and that the operationalization of survey questions leads to a narrow definition of public opinion (McGregor, 2019). Recently, social media networks, such as Twitter, have demonstrated to be major drivers of news dissemination and public discourse. It provides a vast amount of semi-structured data in nearly real time and gives a direct access to contents of conversations (Müller et al., 2019). Whittingham et al. (2020) analyzed Twitter posts that discussed genetically modified organisms (GMOs) and found that personality (individual differences in one’s tendency to show consistent patterns of thinking, emotion, and behavior) and values (learned beliefs about one’s preferred way of action or existence) significantly affected GMO risk perception. Twitter discourse regarding clustered regularly interspaced short palindromic repeats (CRISPR), a genome editing technology, was recently investigated using semantic network analysis (Calabrese et al., 2019) and sentiment analysis (Müller et al., 2019). Therefore, we surmised that Twitter analysis could be applied to collect fresh voice on genome-edited foods because it would be possible to quickly collect information on the aspects that people are concerned about or interested in with regard to changes in the situation surrounding genome-edited foods.

2019 was a milestone year for the regulation of genome-edited products in Japan. The Ministry of Environment and five other ministries released policies for handling genome-edited organisms (Tabei, 2019b). As for labeling of genome-edited foods, on June 20, in a subcommittee meeting, the Cabinet Office’s Consumer Committee implied that mandatory labeling for genome-edited products would be difficult (Cabinet Office’s Consumer Committee, 2019). On September 19, the Ministry of Health, Labour and Welfare (MHLW) published a notification on genome-edited foods, announcing that it would be implemented from October 1 onward (Ministry of Health, Labour and Welfare, 2019). At the same time, the Consumer Affairs Agency (CAA) revealed their policy on voluntary labeling of genome-edited foods. There was also much discussion about the labeling of GM crops. Consumers demanded clear labeling to guarantee transparency, and in 2001, the Ministry of Agriculture, Forestry and Fisheries (MAFF) and the MHLW issued a labeling policy that lead to mandatory labeling of GM foods if the transgene can be detected (ISAAA, 2006). With regard to the commercialization of genome-edited foods, labeling is likely be a prevalent discussion. So, it seemed important to evaluate how the general public expressed their opinions and responded before and after the labeling policy was announced. Therefore, we decided to narrow our Twitter analysis to tweets related to the labeling of genome-edited foods. In this study, we collected Japanese Twitter data and investigated the relation between government announcements and news published by media. Furthermore, the appearance of words in tweets was investigated using co-occurrence networks, and emotions were determined using sentiment analysis. From these results, the use of Twitter analysis for knowing users’ opinions with regard to genome-edited foods and their labeling and its potential contribution to the communication strategy toward social implementation was discussed.

## Materials and Methods

### Collecting Twitter Data

To explore public concern regarding genome-edited foods and their labeling, Twitter data were collected from a cloud software, Mieruka Engine^®^ (Plus Alpha Consulting Co., Ltd., Tokyo, Japan), from May 25 to October 15, 2019, with the search string “genome editing (which included ‘genome-edited’ in Japanese) AND labeling” in Japanese. We performed Mieruka Engine^®^ searches using API provided by the NTT DATA Corporation (Tokyo, Japan), which has the resale rights of Twitter data in Japan. The use of materials for data analysis is permitted by the Copyright Act of Japan.

### Refining Twitter Data

There are three types of tweets: original tweets, reply tweets, and retweets. In our study, retweets were regarded as partially or fully agreeing with the opinions of the original tweeters. Although tweet redundancy increases the chance of opinions influencing many people, we excluded duplicates for an accurate analysis. The collected tweet data were refined according to the following rules: (i) if a Twitter user tweets or retweets the same text multiple times, whether intentional or not, only one of them is analyzed; (ii) if the duplicate tweets were from different people, those texts are all subject to analysis – this happens frequently in retweets; and (iii) if a tag or link is different but the text is the same, it is considered the same tweet.

### User Profile Data

Twitter users registered their profiles when creating their accounts; some left them blank. For users without profiles, Mieruka Engine^®^ predicted their profiles when possible. Unique user IDs posted from May 25 to October 15 were collected and counted. Because of system rules, user profiles of tweets from June 15 to October 15 were available in Mieruka Engine^®^. To verify whether the same users tweeted repeatedly on this issue, unique user IDs were categorized based on tweet counts (including original, reply tweets, and retweets) posted during this period.

### Tweet Peak Detection

During data collection, the tweet count (i.e., original, replies, and retweets) increased sharply on several occasions. Prominent peaks were detected with Python (version 3.7.6), using SciPy 1.4.1. The prominence of a peak allows measurements of the degree to which a peak is protruding, depending on its position in relation to other peaks. A prominence cut-off of 300 was set to detect important peaks evenly over the entire period of the study. When peaks were detected, influential events were determined from the content of each tweet.

### Analysis of Appearance Pattern of Words in Tweets by Co-occurrence Network

On September 19, the MHLW announced that the notification of genome-edited foods would start on October 1, and the CAA declared that labeling of genome-edited foods should not be mandated. To examine the overall picture of how users discussed genome-edited foods and their labeling in response to government’s announcements, we created co-occurrence networks based on tweets posted from September 19 to 22. At first, we analyzed 530 original and reply tweets excluding retweets using the free text-mining software KH Coder (Higuchi, 2016). Then we analyzed all 5,410 tweets by the same method described above. Before analysis, we modified the text data of the tweets as follows: (i) we converted half-width Japanese characters to full-width Japanese letters, full-width numbers and English letters to half-width numbers and English letters, respectively; (ii) we excluded URLs and the string of ASCII characters that are considered punctuation characters; and (iii) we normalized Unicode strings. We also excluded some words as stop words (Supplementary Table 1). We determined the degree of association between words using the Jaccard coefficient (Romesburg, 1992).

### Sentiment Classification of User’s Opinion on Genome-Edited Foods and Labeling

To infer users’ opinions on these policies, original and reply tweets posted during the same period in the former section were selected for classification according to sentiment on genome-edited food and its labeling. Oftentimes, the sentiments expressed for “genome-edited food” and “labeling” in tweets differed; therefore, they were counted independently, and text and information provided by the URL link were used to determine the sentiments. Three researchers differentiated Twitter sentiment into three groups: positive, negative, and neutral. Tweet classification was performed using the criteria in Table 1. In addition, the sentiment of each tweet in the same data set was also determined using the “positive–negative analysis” function of the Mieruka Engine^®^ software.

**TABLE 1:** Tweet classification criteria for sentiment analysis.

Sentiment	Criterion
Positive	This category includes tweets that accept genome-edited food or government policies, explain the technology scientifically, or elucidate the reason for non-mandatory labeling.
Negative	This category consists of tweets against genome-edited foods and MHLW/CAA policies or those that call for a signature petition of severe regulation. Many of them include the following terms: “scary,” “don’t want to be distributed,” “right not to eat,” “don’t want to buy,” “don’t want to eat,” “dangerous,” etc.
Neutral	This category includes tweets that are neutral on genome-edited food/labeling or are just publicizing government policies, etc.

## Results

### Tweet Data and User Profiles

From the 29,299 tweets that were extracted with the search string “genome editing (which included ‘genome-edited’ in Japanese) AND labeling” in Japanese, 577 were excluded in accordance with the policies laid out in the “Materials and Methods” section. Thus, the dataset consisted of 28,722 tweets of which 2,536 tweets (8.8%) were original tweets, 326 tweets (1.1%) were replies, and 25,860 tweets (90%) were retweets.

To learn about the kind of people who were interested in the labeling of genome-edited foods, we categorized the age and gender of unique user IDs (Table 2). Of 14,066 unique user IDs, gender and age profiles were available for 12,016. While user profile accuracy is not guaranteed, 8,817 (73.4%) were male, and 3,199 (26.6%) were female. As for age distribution, the ratio of users in their 10s and 40s were low, accounting for only 2.3% and 11.3%, respectively, while, users in their 30s and 50s and above were relatively high, at 25% and 45.6%, respectively. In particular, users in their 50s and older were the largest group among male users while users in their 30s were the largest group among female users (Table 2).

**TABLE 2:** Unique user IDs categorized by age and gender.

Age	Gender				Total	
		
	Male		Female			
10s	168	(1.9)	110	(3.4)	278	(2.3)
20s	1,270	(14.4)	632	(19.8)	1,902	(15.8)
30s	1,748	(19.8)	1,254	(39.2)	3,002	(25.0)
40s	834	(9.5)	524	(16.4)	1,358	(11.3)
50s and over	4,797	(54.4)	679	(21.2)	5,476	(45.6)
Total	8,817	(100.0)	3,199	(100.0)	12,016	(100.0)

*The numbers on the left represent the number of unique user IDs of all tweets posted from June 15 to October 15. The numbers in parentheses indicate percentage.*

To verify that the same users tweeted repeatedly in each peak, unique user IDs were categorized based on tweet counts (Supplementary Figure 1). The study found that 94.9% of user IDs posted five or less tweets, in particular 64.8% and 17.3% posted tweets only once and twice, respectively, whereas 94.9% posted five or less tweets. Two user IDs were noted to post more than 100 tweets, and the maximum tweet count per user ID was 201.

### Changes in Tweet Count and Influential Events

**FIGURE 1:**
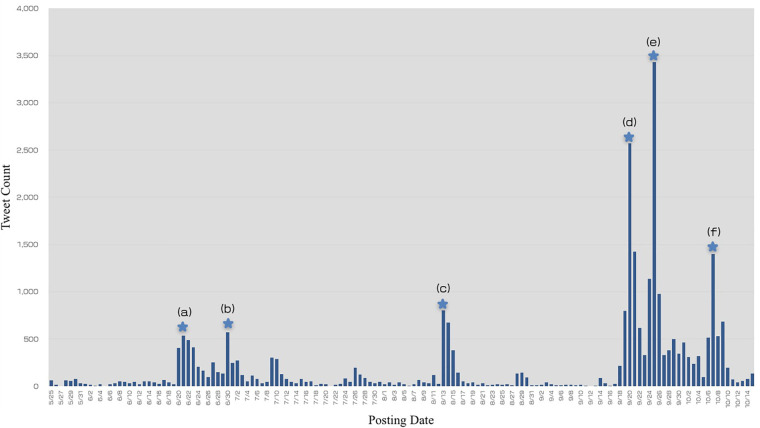
Changes in the daily number of tweets. This figure shows the number of tweets in chronological order from May 15 to October 15. The bars represent the total tweet count including original tweets, reply tweets, and retweets. Prominence of peaks (a–f) were identified with Python (version 3.7.6) using the SciPy 1.4.1.

We investigated the time course of daily tweet numbers to reveal the responses to information related to the labeling of genome-edited foods. Figure 1 shows the change in the daily number of tweets from May 25 to October 15. The tweet count ranged from 1 to 3,426 a day. During this period, we also identified six specific peaks. Table 3 summarizes the date and tweet count in the peaks as well as influential events (e.g., government announcements, media reports, and so on).

**TABLE 3:** Changes in tweet counts in response to influential events.

Peak^*a*^	Date	Influential events^*b*^	No. of all tweets	No. of original and reply	No. of retweets	Prominence^*c*^
(a)	6/21	A subcommittee meeting of the Cabinet Office’s Consumer Committee stated that it is difficult to make the labeling of genome-edited foods mandatory on June 20.	535	54	481	436
(b)	6/30	The Ministry of Health and Welfare (MHLW) began to collect public comments on genome-edited foods from June 27.	569	13	556	566
(c)	8/13	An opposition politician posted a tweet on August 13 insisting mandatory labeling of genome-edited foods.	802	13	789	799
(d)	9/20	The MHLW and the Consumer Affairs Agency announced their policies for handling and labeling genome-edited foods on September 19.	2,569	275	2,294	2,239
(e)	9/25	Many news introducing the basis of genome editing technology and reviewing government’s policies were widely publicized in TV programs, newspapers, and so on.	3,426	142	3,284	3,387
(f)	10/7	There seemed to be no major events, but there were various kinds of tweets on news, signature activities, and so on.	1,397	129	1,268	1,297

*^*a*^The first column represents the peaks in Figure 1. ^*b*^Influential events include government announcements, mass media, and tweets by influential public figures. ^*c*^The prominence of each peak is calculated using SciPy 1.4.1, which is module of Python.*

When it was expressed in a subcommittee meeting of the Cabinet Office’s Consumer Committee that mandatory labeling for genome-edited foods would be difficult, many users posted tweets in response to news regarding it. This is a key reason for the significant increase in tweet count compared to the period before June 21 and thus formed the peak in Figure 1(a). The MHLW then began collecting public comments on the procedure for submitting information on genome-edited foods (Ministry of Health, Labour and Welfare, 2019). Many media networks covered the direction of notification, which was a trigger to form the peak in Figure 1(b).

The third peak, Figure 1(c), several tweets mentioned a tweet by an opposition politician insisting mandatory labeling and tweets introducing newspaper or website articles, and most tweets were about campaigns to collect signatures demanding the mandatory labeling of genome-edited foods.

The fourth peak, Figure 1(d), shows that the tweet count rose immediately after the government announced its handling policies on genome-edited foods on September 19. The MHLW announced that starting October 1, it would be requiring notification, not safety assessment, for genome-edited foods before commercialization. The CAA, meanwhile, announced that the labeling policy for genome-edited foods was non-obligatory but recommended that developers provide as much information to consumers as possible. News about the government’s policies were publicized by many media outlets over 2 days from September 19 to 20. The increase in tweet count on September 20 appears to be because of widespread tweeting. Many tweets tried to publicize the fact that genome-edited food labeling is not mandatory, of which a few were positive opinions. Examples include “It is reasonable that labeling of genome-edited food is voluntary, as it is scientifically indistinguishable from existing food,” and “It’s not good to just look at the word genome editing and post negative messages.” However, there were also many negative opinions expressing concern that one could unwittingly buy genome-edited foods.

The fifth peak, Figure 1(e), was mainly caused by much news that introduced genome editing technology and reviewed government’s policies, which were widely publicized in TV programs, newspapers, and so on. These included a special TV program aired by the Japan Broadcasting Corporation (NHK), editorial articles, and others. There were also many tweets about petitions to collect signatures for mandatory labeling. The tweet count on September 25 was 3,426, the highest number during the analysis period.

The cause of the sharp increase in tweet count on October 7, the peak in Figure 1(f), was unclear. No major event seemed to have occurred, but there were various kinds of tweets regarding news about labeling, signature activities that oppose to non-obligatory labeling, and so on. Moreover, a newsletter article posted on a bulletin board system (BBS), the largest in Japan, was tweeted and retweeted. This BBS often discusses opposition to social trends and events that have not spread to society.

### Words in Tweets by Co-occurrence Network

Using co-occurrence networks, we analyzed the appearance and relevance of words in original and reply tweets, and all tweets including retweets posted from September 19 to 22 and compared patterns of the two networks.

**FIGURE 2:**
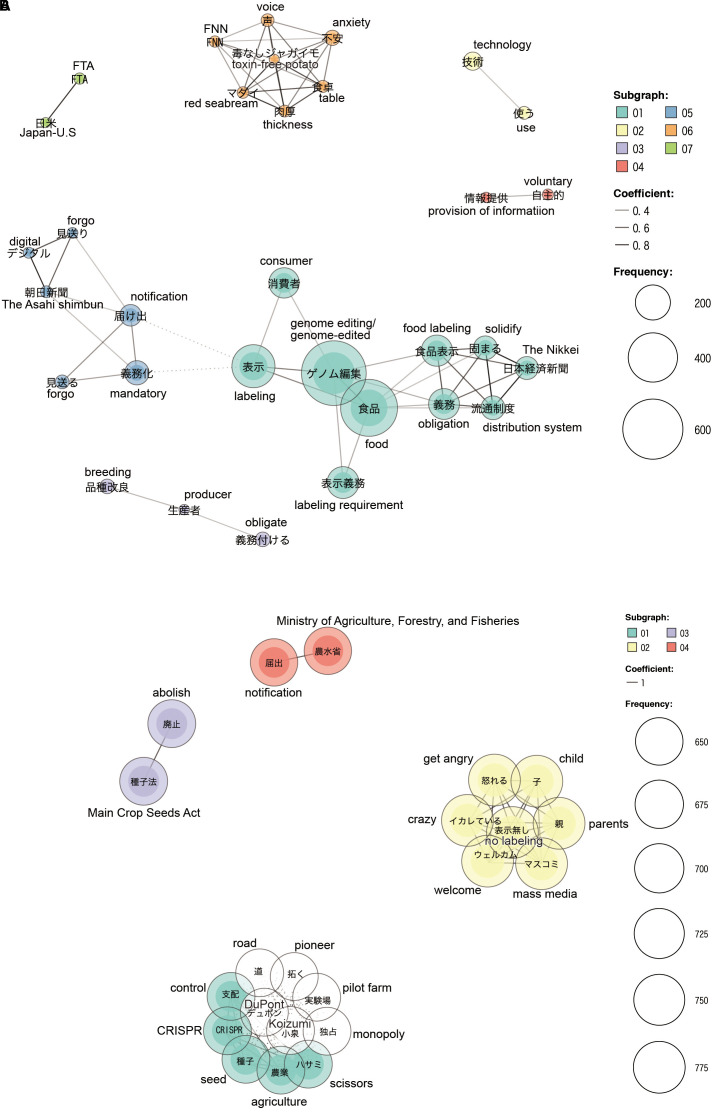
Co-occurrence networks of words in tweets posted from September 19 to 22. **(A)** Co-occurrence network with only original and reply tweets, **(B)** co-occurrence network with all tweets including original tweets, reply tweets, and retweets.

In the co-occurrence network of original and reply tweets without retweets, seven clusters were observed (subgraphs 1–7; Figure 2A), which had the following characteristics: Subgraph 1 consisted of words mainly related to *genome-edited*, *food*, *mandatory labeling*, and *consumer*, which were discussed in the context of the obligatory labeling of genome-edited foods. Furthermore, the word *the Nikkei* (referring to the *Nikkei* newspaper) was strongly related to the words *food labeling, obligation*, and *distribution system*; it was speculated that the content published in the *Nikkei* uses the above words. Subgraph 2 contained two words: *technology* and *use*. Subgraph 3 consisted of three words – *breeding*, *producer*, and *obligate* – which points toward people asking producers for mandatory labeling. Subgraph 4 consisted of two words: *voluntary* and *provision of information*. It shows that many tweeters were concerned about the provision of information (in the notification to the MHLW) being non-mandatory. Subgraph 5 consisted of tweets regarding news of government policies and the commercialization of genome-edited foods, and an article from *Asahi Shimbun* (Asahi newspaper) influenced this subgraph. Subgraph 6 was influenced by tweets regarding studies on potatoes without harmful substances and fast-growing red sea breams, as well as tweets about the resulting public anxiety. Subgraph 7 contained two words: *Japan and United States* and *FTA*. Tweeters were concerned that the United States–Japan FTA might result in the import of undesirable genome-edited foods because of pressure from the United States.

However, in the co-occurrence network of all tweets, two large clusters consisting of 12 and 7 words and two small clusters containing two words were formed. Concerns about food supply and Japanese agriculture formed a cluster (subgraph 1, Figure 2B); which included words such as *seed*, *agriculture*, *control*, and *CRISPR.* In addition, these words were also co-occurring with words in subgraph 1, such as *DuPont*, *monopoly*, *pilot farm*, and *Koizumi*, a member of the Japanese Diet, i.e., Japanese parliament, regarded as a key player in abolishing the Major Crop Seeds Act. Representative contentions of the tweets related to this cluster were “CRISPR-Cas9 is a carcinogenic enzyme,” “the abolition of Major Crop Seeds Act would cause the control of Japanese agriculture by foreign-affiliated companies,” “promotion of local production for local consumption,” and so on. These words were associated with the concern that foreign companies would control or monopolize crop seeds. Another cluster (subgraph 2, Figure 2B) was formed by the users’ anger toward the CAA’s policy of not imposing mandatory labeling, and concerns about children’s health and food safety. The terms in this cluster were *mass media*, *get angry*, *no labeling*, *parents*, *child*, *crazy*, and *welcome*. The word *welcome* was used in the context of criticizing news which welcomed genome editing technology, and does not pertain to agreement with the CAA policy. Other small clusters contained such terms as *Main Crop Seeds Act*, *abolish*, *the Ministry of Agriculture*, *Forestry and Fisheries*, *notification*, and so on. Tweets with such words seemed opposed to genome editing or to the government’s agricultural policies.

The co-occurrence network of original and reply tweets provided different words from various tweets that appeared with a certain frequency, and many of them were derived from news contents. However, the network derived from all tweets seemed to concentrate on words from specific tweets with negative overtones, and the variety of words that appeared in the network was biased, resulting in a simple network diagram.

### Sentiment Analysis of Tweets on Genome-Edited Food and Labeling

Five hundred and thirty original and reply tweets posted during 4 days after the government announcements from September 19 to 22 were classified as positive, negative, and neutral based on sentiments on genome-edited food and labeling (Table 4). Three researchers performed the classification. For some tweets, Twitter’s character limit (140 Japanese characters) and chatty and colloquial style of tweets complicated sentiment judgment. In instances where the classification of a given tweet among the three researchers was inconsistent, the sentiment agreed by two of them was adopted. When there were three different judgments for a tweet, it was classified as neutral. There were 190 (35.8%) and 122 (23.0%) tweets indicating inconsistent judgment among three researchers for genome-edited foods and labeling, respectively.

**TABLE 4:** Types of sentiments on genome-edited food and its labeling expressed in each tweet posted from September 19 to 22, 2019^*a*^.

		Labeling			
		Positive	Negative	Neutral	Total^*b*^
Genome-edited food	Positive	24	8	5	37 (7.0)
	Negative	0	286	3	289 (54.5)
	Neutral	3	39	162	204 (38.5)
	Total^*b*^	27 (5.1)	333 (62.8)	170 (32.1)	530 (100.0)

*^*a*^Tweet classification was performed by three researchers using the criteria in Table 1. ^*b*^The numbers in parentheses indicate percentage.*

There were tweets in which users tried to convey scientific knowledge to other users, such as explaining the difference between genome editing and genetic modification, and such tweets were classified as “positive.” Tweets that include phrases such as “want to eat” and “want to buy” were also under “positive.” Under the “negative” classification were tweets against MHLW/CAA policies, those that call for a signature petition for stricter regulations, and so on. Tweets that mention articles that clearly criticize the government’s policies were also classified as “negative,” Most “neutral” tweets were those that simply publicized news by mass media networks.

Of the 530 original and reply tweets posted during the period, 289 (54.5%) opposed genome-edited food, and 37 (7.0%) were in favor of it. With regard to the CAA policy, 333 (62.8%) tweets were negative and 27 (5.1%) were positive (Table 4). Among the 37 positive tweets about genome-edited food, 24 were in favor of the CAA policy and 8 were against it, for example, “It is better that it is labeled, because I want to buy and eat it.” 204 tweets (38.5%) were neutral toward genome-edited food, but 39 opposed the non-mandatory labeling; for instance, “Aside from the discussion on whether genome-edited foods are safe, its labeling is necessary for consumers to have a choice.”

For comparison, the sentiment for each tweet was determined using the Mieruka Engine^®^ software, which did not consider the respective sentiments for genome-edited food and labeling policy in a tweet. Of the same 530 tweets mentioned above, 79 tweets (14.9%) were determined to be positive, while 97 (18.3%) were determined to be negative.

## Discussion

Genome editing technology is expected to rise in fields such as medicine and agriculture. However, science and technology are not autonomous entities, and research trajectories are largely influenced by public opinion; even if crops produced using new breeding techniques do not fall under GMOs, commercializing them is by no means easy (Ishii and Araki, 2016; Malyska et al., 2016). The public tends to have a vague anxiety about advanced technology that they are not familiar with. Therefore, public acceptance of innovative technology, such as genetic modification or genome editing, is an important requirement for its social implementation. In the 1990s, when GM foods began to be distributed in Japan, there was much debate about labeling to ensure consumers’ right to select non-GM foods. As a result, the MAFF and MHLW started the labeling system for GM food in 2001 (ISAAA, 2006; Umeda, 2014). It was assumed that labeling would be a major issue for the social implementation of genome-edited foods; therefore, Twitter analysis relevant to genome-edited foods and their labeling was started.

In this survey, among user IDs whose profiles were available, 71.5% were male, and male users in their 50s and older outnumbered male users in other age groups (Table 2). Females in their 30s were the most among all age groups of female users. Generally, Twitter users are relatively evenly distributed across all ages although the number of males in their 40s and females in their 20s tend to be slightly more than other ages^2^. The reason for the deviation in our study was not clear, but users with these profiles were most interested in genome-edited foods and their labeling. A total of 94.9% of user IDs posted five or less tweets, in particular 64.8% tweeted only once. The majority of the users who tweeted about this issue do not seem as intense as they posted tweets consistently.

The study retrieved a total of 29,299 tweets from May 25 to October 15 in 2019 (142 days) using the search string “genome editing (which included ‘genome-edited’ in Japanese) AND labeling” in Japanese with a maximum tweet number per day of 3,426. To compare the number of tweets on this issue and another much-discussed political issue, tweets on a political issue (i.e., the Tokyo gubernatorial election) were retrieved using the Mieruka Engine^®^ from June 29 to July 29, 2020. The maximum tweet count per day reached 1,343,045 tweets on the election day: June 5. In terms of the Fukushima Daiichi Nuclear Power Plant accident, which was directly related to life-threatening and energy problems, approximately 25 million tweets were posted within a span of 195 days, and the maximum tweet count per day was more than 640,000 (Tsubokura et al., 2018). Comparing these politically and socially hot topics, the issue on labeling of genome-edited food seemed to draw less attention on Twitter. Typically, surveys on public opinion are conducted and targeted several thousands of samples, which are nationally representative. In the present study, online discourse and extent of users’ interest on the labeling of genome-edited food were successfully obtained, although bias in Twitter users or limited number of tweets posted on this issue should be considered. To collect opinions from a diverse or targeted range of people, questionnaire surveys should be used together.

To ascertain users’ opinions with regard to the labeling of genome-edited foods, Twitter data were collected from May 25 to October 15 using the search string “genome editing (which included ‘genome-edited’ in Japanese) AND labeling.” Throughout this period, the tweet count changed significantly in response to government announcements and news published by the media and other information sources. A spike in the tweet count may be an indication of growing concern among Twitter users (Figure 1 and Table 3). An analysis of the sentiments accompanying original and reply tweets revealed that 289 tweets (54.5%) displayed negative sentiments toward genome-edited foods. Uchiyama et al. (2019) reported the results of an attitude survey on the internet that showed that 43% and 47% of Japanese citizens answered that they did not want to eat genome-edited crops and livestock products, respectively, similar to the results of sentiment analysis in present study. To reveal the reason for this evasion, a more comprehensive analysis of opinions regarding genome-edited foods and the application of genome editing technology to food would be necessary while using a data set not limited to labeling. With regard to the non-mandatory labeling policy of the CAA, 333 tweets (62.8%) opposed it. Sentiments for genome-edited foods and their labeling policy displayed different tendencies. All negative tweets about genome-edited foods were negative or neutral about the labeling policy. However, among tweets that were positive about genome-edited foods, some tweets were negative about the labeling policy. Therefore, the percentage of negative tweets about labeling policy was higher than that for genome-edited foods itself, indicating a strong demand for mandatory labeling, as was the case of GM crops of which labeling was requested by consumers because they had doubts about its safety and wanted to avoid GM foods (Umeda, 2014). These findings highlighted the need to inform people regarding the rationale behind the CAA policy of non-mandatory labeling. A technical reason exists for the non-mandatory labeling of genome-edited foods, because distinguishing between genome-edited foods and those developed using conventional breeding is difficult.

Sometimes, the Twitter character limit (140 characters) and the chatty, colloquial style of tweets complicated sentiment judgment, resulting in approximately one-quarter to one third inconsistent tweets among the three researchers. Sentiments determined by the Mieruka Engine^®^ software tended to be positive more than those determined by researchers in this study. Sentiment analysis of the software was conducted by determining the presence of words in positive and negative categories, and not by the context. This might be a reason for the difference between the two methods. For high-volume and automated processing, the deep learning of text data could potentially allow for the identification of complex sentence structures, taking into account multiple word combinations, and lead to improvement in the accuracy of sentiment analysis.

Retweets accounted for 90% of total tweets in our study; co-occurrence networks were significantly different with or without retweets because of an increase of the proportion of specific words in retweets (Figure 2). There are several finding on retweets. Boyd et al. (2010) found that people prefer retweets that contain breaking and timely news. Naveed et al. (2011) found that tweets that address public events or include emoticons that reveal more negative emotions than positive ones are likely to be retweeted; Negative tweets are generally more often retweeted than positive ones regardless of their subject matter (Stieglitz and Dang-Xuan, 2012; Tsugawa and Ohsaki, 2015). These findings are consistent with our results on co-occurrence networks that words from tweets with a negative tone increased when adding retweets to the analysis. Such retweets, which were made up majorly of tweets obtained in our study, might be a product of the increasing disaffection and anxieties of users about the labeling policy. The negative opinions of users, revealed by a co-occurrence network and sentiment analysis, seem to imply the direction of communication strategy of genome-edited food.

The acceptance of GM crops increased among consumers who understood the benefits of GM crops, such as reduced CO_2_ emissions (Council for Biotechnology Information Japan, 2017). For some genome-edited crops that are currently being developed in Japan, it is expected that consumers easily understand their benefits; some examples of such crops are high-GABA tomatoes (Nonaka et al., 2017) and potatoes without harmful substances (Sawai et al., 2014). It might be easier to achieve public acceptance and to realize social implementation for such crops. This study indicated the potential of Twitter as a real-time indicator of users’ concern. When releasing genome-edited crops into the Japanese market, it is likely that there will be discussions about them pros and cons, and it would be significant to continue analyzing opinions for them.

## Conclusion

Twitter analysis allowed us to quickly access online discourse in response to government announcements and media reports. Through this study, we were able to identify the information that Twitter users were interested in and also their apprehensions with regard to genome-edited foods and their labeling. These findings may contribute to the communication strategy of genome-edited foods with regard to its social implementation, for example, in case erroneous information spreads, corresponding scientific facts could be provided. However, there are certain disadvantages of Twitter analysis such as the limit on information that can be derived from each tweet due to the character limit (140 Japanese characters), colloquial style, and bias among Twitter users. To achieve a more detailed and accurate analysis, using a combination of a Twitter analysis and other methods, such as questionnaire surveys, would help cover for the deficits of the Twitter analysis. Furthermore, it will be meaningful to analyze consumers’ opinions and attitudes at the first commercialization of genome-edited food employing data mining by deep learning, considering many complex factors, such as social situation surrounding genome-edited foods, news content, online discourse or questionnaire survey, and individual profile, among others, in the future.

## Data Availability Statement

All dataset except raw Twitter data generated for this study are included in the article/Supplementary Material.

## Author Contributions

YT provided the initial research design, discussed the results, and wrote, reviewed, and edited the manuscript. SS wrote the manuscript, performed research design and data analysis, and discussed the results. YK also wrote the manuscript and worked on research design, data collection and analysis, and the discussion. SI discussed the results as well. NF worked on data analysis and discussed the results as well as reviewed and edited the manuscript. All authors contributed to the article and approved the submitted version.

## Conflict of Interest

The authors declare that the research was conducted in the absence of any commercial or financial relationships that could be construed as a potential conflict of interest.

## Footnotes

1 https://calyxt.com/first-commercial-sale-of-calyxt-high-oleic-soybean-oil-on-the-u-s-market/

2 https://gaiax-socialmedialab.jp/post-30833/
