# Supplementary figures and images for: Analyzing Twitter Conversation on Genome-Edited Foods and Their Labeling in Japan
Source: Front Plant Sci. 2020 Oct 22;11:535764. doi: 10.3389/fpls.2020.535764 (PMC7642521; doi:10.3389/fpls.2020.535764)

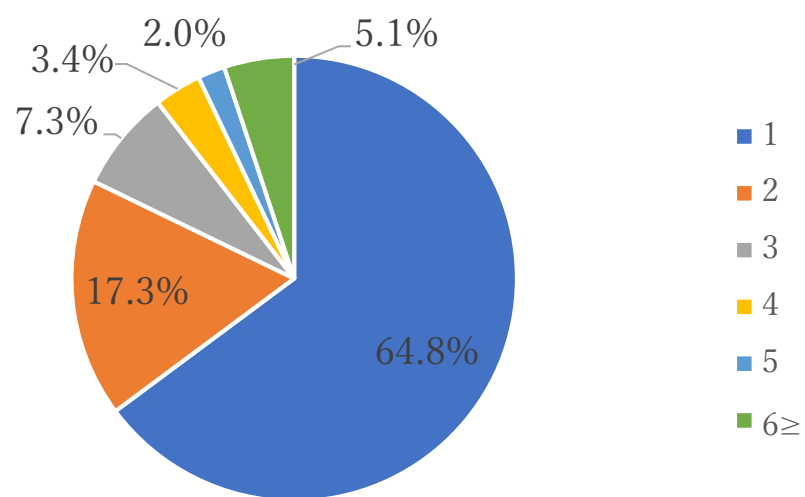

Supplementary Figure 1. Percentage of average tweet counts per user ID across the survey period.

Supplement: Supplementary file 1 [file Image_1.pdf]
