# Supplementary material for: Analyzing Twitter Conversation on Genome-Edited Foods and Their Labeling in Japan
Source: Front Plant Sci. 2020 Oct 22;11:535764. doi: 10.3389/fpls.2020.535764 (PMC7642521; doi:10.3389/fpls.2020.535764)
Supplement: Supplementary file 2 [file Table_1.pdf]

Supplementary Table 1. Stopwords

Type of Words	Stopwords
Letters	Meaningless English/Japanese words, single characters, and single Chinese characters that are meaningless or related to a day of week
Proper nouns	Names, RT+UserID, title (e.g. minister), Place names
Verbs	“be”, “think”, “do”, “say”, “want” and, inflected forms of these verbs
Other parts of speech	Pronouns, demonstrative pronouns, interjections, suffixes, prefixes, connectives, temporal nouns, Japanese abstract nouns （e.g. “koto”,”no”,”mono”）
Specific words	Please RT, news, regulation, duty, campaigns, signatures, activities, other, by chance, results, finally, fact

Notes: Original Stopwords were Japanese.

Specific words were excluded from the analysis because they appeared frequently in tweets and could have become impeditive factors.
